# Enhancing Antimicrobial Performance of Gauze via Modification by Ag-Loaded Polydopamine Submicron Particles

**DOI:** 10.3390/jfb15060152

**Published:** 2024-06-02

**Authors:** Junnan Cui, Haobo Shu, Panpan Zhu, Zhimin Cao, Shuilin Wang, Pan Cao

**Affiliations:** 1 School of Mechanical Engineering, Yangzhou University, Yangzhou 225127, China; mx120230545@stu.yzu.edu.cn (J.C.); mz120220919@stu.yzu.edu.cn (H.S.); zppjqb@163.com (P.Z.); 2 Institute of Intelligent Manufacturing and Smart Transportation, Suzhou City University, Suzhou 215104, China; wsl2023@szcu.edu.cn

**Keywords:** Ag-loaded PDA submicron particles, modified gauze surface, antimicrobial performance, cell compatibility

## Abstract

Silver nanoparticles (AgNPs) are known for their antibacterial properties and their ability to promote wound healing. By incorporating silver nanoparticles into medical gauze, the resulting composite material shows promise as an advanced wound dressing. However, clinical applications are hindered by challenges related to the stability of silver nanoparticle loading on the gauze as nanoparticle leaching can compromise antibacterial efficacy. In this study, silver nanoparticles were immobilized onto polydopamine (PDA) submicron particles, which were then used to modify medical gauze. Energy dispersive spectroscopy (EDS) was employed to analyze the elemental distribution on the modified gauze, confirming successful surface modification. The antibacterial properties of the modified gauze were assessed using a laser scanning confocal microscope (CLSM). The results demonstrated a significant reduction in the adhesion rates of *Escherichia coli* (*E. coli*) and *Staphylococcus aureus* (*S. aureus*) by 99.1% and 63%, respectively, on the PDA–Ag-modified gauze. Optical density (OD) measurements at 590 nm indicated that the modified gauze effectively inhibited biofilm formation, underscoring its potent antimicrobial capabilities. Further antibacterial efficacy was evaluated by diluting and plating co-cultured bacterial solutions with the modified dressing, followed by 24 h incubation and colony counting. The gauze exhibited an antibacterial efficiency of 99.99% against *E. coli* and 99.8% against *S. aureus*. Additionally, cell compatibility tests, involving the co-culture of PDA–Ag composites with human cells, demonstrated excellent biocompatibility. These findings suggest that PDA–Ag-modified medical gauze holds significant potential for the treatment of infected wounds, offering a promising solution to improve wound care through enhanced antimicrobial activity and biocompatibility.

## 1. Introduction

Skin injuries have significant implications for human well-being and present a complex challenge in the medical field [1,2]. Medical gauze, a commonly employed material in hospitals, is traditionally manufactured from cotton fibers. The selection of cotton is based on its advantageous properties, including softness, moisture absorption capacity, and cost-effectiveness, making it a popular choice for wound dressing applications. However, the porous structure and water-absorbing nature of cotton gauze create an environment conducive to the proliferation of pathogenic microorganisms, increasing the risk of wound-associated cross-infections and hindering the healing process [3,4,5]. This issue is exacerbated by the emergence of antibiotic-resistant bacterial strains, leading to severe wound infections and impaired healing outcomes [6]. In light of the escalating concern over medically significant infections, there is an urgent need to develop advanced medical gauze formulations capable of effectively preventing wound infections and promoting the healing process [5,7,8].

There have been extensive studies aimed at conferring antibacterial properties to cotton fabrics through the incorporation of various antibacterial agents, including antibiotics [7] and metal nanoparticles [8]. Ideally, upon contact with a surface containing an antimicrobial agent, pathogens are targeted, leading to their eradication. Silver nanoparticles, known for their antimicrobial efficacy against drug-resistant bacteria [9,10,11], primarily function by interacting with bacterial enzymes and proteins, as well as disrupting bacterial membranes [12]. Despite their potent antimicrobial activity, individual silver nano-particles are prone to encapsulation by adsorption-conditioned membranes composed of proteins and deceased microorganisms, thereby diminishing their antimicrobial efficacy [13]. Dopamine, a catecholamine molecule, possesses strong cell adhesion properties, excellent biocompatibility, and structural similarities to the foot protein Mefp-5, demonstrating low toxicity to human cells [14,15]. The abundance of reactive catechol and amine groups on dopamine’s surface enables its adhesive polyamine coatings to serve as effective reducing agents and binders, facilitating the synthesis of versatile composites with tailored functional properties [16]. Recent advancements have seen dopamine combined with metal ions, such as silver ions, leveraging its robust adhesion capabilities to synthesize silver nanoparticles in situ on diverse substrate materials [17]. However, the clinical application of silver-modified cotton dressings is impeded by the leaching of silver nanoparticles from the cotton fiber surface. To address this challenge, silver nanoparticles are modified with polydopamine (PDA) submicron particles.

**Figure 1:**
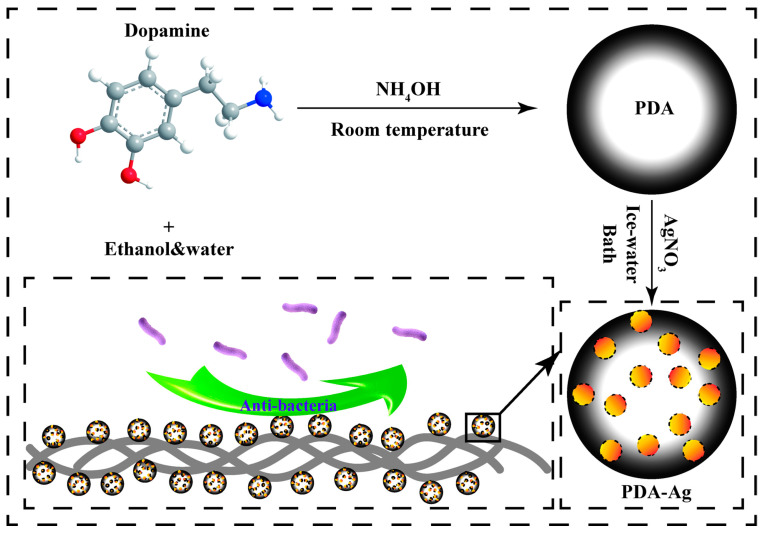
A modified antimicrobial gauze surface synthesis is visually demonstrated in a schematic illustration.

In this study, polydopamine nanosilver composites were synthesized in a one-pot reaction utilizing polydopamine spheres as both reducing and stabilizing agents. Leveraging the unique properties of dopamine, the particle size of sub-micron polydopamine particles was optimized by manipulating various factors. Subsequently, the synthesized composites were immobilized onto medical gauze, as illustrated in Figure 1. Through a comprehensive series of biotoxicity and antimicrobial assessments, the modified gauze exhibited robust bacteriostatic adhesion properties, effectively impeding biofilm formation and demonstrating promising prospects for practical applications.

## 2. Materials and Methods

### 2.1. Materials

Dopamine hydrochloride, propidium iodide (PI), 2-Methylimidazole, glutaraldehyde (50%), and anhydrous ethanol were procured from Aladdin Reagent Co (Shanghai, China). Sodium chloride, silver nitrate, crystal violet, and glacial acetic acid were sourced from Sinopharm Chemical Reagent Co (Shanghai, China). All chemicals used in this study were of analytical grade. Sterile medical gauze was obtained from Yangzhou Yurun Science and Technology Development Co., Ltd., while ammonia (28–30%) was acquired from Shanghai McLean Biochemical Science and Technology Co., Ltd. Phosphate buffer solution (PBS, pH = 7.4) was purchased from Beijing Wokai Biotechnology Co. (Beijing, China), and LB liquid medium, as well as LB solid medium, were obtained from Qingdao Hi-Tech Industrial Park HaiBo Biotechnology Co., Ltd (Qingdao, China). HEK293T cells were primarily cultured in Dulbecco’s modified Eagle medium (DMEM, Thermo Fisher Scientific, Waltham, MA, USA) supplemented with 10% fetal bovine serum (FBS, Cytiva, Dhaka, Bangladesh) at 37 °C in a 5% CO_2_ environment in an incubator.

### 2.2. Synthesis of Polydopamine Submicron Particles

At the onset of the experiment, deionized water (90 mL) was meticulously blended with ethanol (40 mL) to ensure precise measurements. This mixing procedure was carried out at room temperature. Subsequently, ammonia solution (NH_4_OH, 2 mL, 28–30%) was gently introduced into the mixture, followed by stirring for 30 min. Dopamine hydrochloride (0.5 g) was dissolved in deionized water (10 mL) and incorporated into the mixture. The resulting solution was divided into two sets, with one undergoing a 30 h reaction and the other a 48 h reaction. The obtained products were subjected to centrifugation for 15 min at 7000 r/min, washed thrice, and subsequently freeze-dried to yield polydopamine (PDA), which was then stored in a vacuum desiccator for future utilization.

### 2.3. Preparation of PDA–Ag Submicron Particles

The obtained PDA was dispersed in deionized water and subjected to sonication for 15 min. Subsequently, 0.8 g of silver nitrate was dissolved in 10 mL of deionized water and added dropwise to the PDA dispersion system. The reduction reaction occurred in an ice bath for 10 min with sonication assistance. Following this, the system underwent three wash cycles via centrifugation to obtain PDA–Ag, which was then dried under vacuum at 60 °C for subsequent applications.

### 2.4. Characterization of PDA and PDA–Ag

A micro-infrared spectrometer (Cary 610/670, Varian, Palo Alto, CA, USA) was employed to conduct measurements across a scanning range of 4000 cm^−1^ to 750 cm^−1^, with a spectral resolution of 4 cm^−1^, to analyze the surface functional groups of the samples. Additionally, X-ray photoelectron spectroscopy (XPS, ESCALAB 250Xi, Thermo Scientific, Waltham, MA, USA) was utilized to examine the surface elements and their respective electronic valence states in the samples. Field emission scanning electron microscopy (Gemini SEM 300, Carl Zeiss AG, Jena, Germany) in conjunction with self-contained energy-dispersive X-ray spectroscopy (EDS, Carl Zeiss AG, Jena, Germany) was employed to scrutinize the surface morphology, elemental distribution, and content of the samples, thereby elucidating the structure and composition of the samples. The distribution of silver nanoparticles on PDA–Ag was analyzed using transmission electron microscopy (Tecnai 12, Philips, Amsterdam, The Netherlands). Laser confocal microscopy (Ultra-high-resolution laser confocal microscope, TSC SP8 STED, Leica, Germany) was employed to observe bacterial adherence to the PDA–Ag-modified gauzes and to evaluate the in vitro antimicrobial properties of PDA–Ag. Fluorescence inverted microscopy (AXIOVERT A1, Carl Zeiss AG, Jena, Germany) was utilized to assess cell attachment on the sample surface, thereby evaluating the compatibility of the PDA–Ag-modified gauze with cells.

### 2.5. Preparation of PDA–Ag-Loaded Gauze Surface

To achieve a homogeneous distribution of PDA–Ag submicron particles within the system, 0.1 g of PDA–Ag submicron particles were dispersed in 30 mL of deionized water and subjected to sonication. A sterile medical gauze (Qi Kang) measuring 10 cm × 10 cm was immersed in the prepared solution and allowed to stand at room temperature for 6 h. Subsequently, the soaked gauze was carefully dried in a 60 °C oven to ensure preservation. This drying procedure was conducted to prepare the gauze for subsequent utilization. To investigate the stability and sustained release kinetics of silver, the PDA–Ag-modified medical gauze was exposed to ambient room conditions for 48 h, followed by examination using scanning electron microscopy to assess the retention of PDA–Ag on the gauze.

### 2.6. Bacterial Culture

Previous research has demonstrated the potent antimicrobial efficacy of PDA–Ag. In the context of potential applications in PDA–Ag dressings, this study focused on assessing the practical antimicrobial performance of PDA–Ag dressings against two bacterial strains, namely, *Escherichia coli* (*E. coli*) and *Staphylococcus aureus* (*S. aureus*). These bacterial strains, representing Gram-negative and Gram-positive bacteria, were procured from the China Medical Culture Collection (CMCC) under the designations CMCC (B) 26003 and CMCC (B) 44102, respectively. To prepare bacterial suspensions for experimentation, isolated colonies of *Escherichia coli* and *Staphylococcus aureus* were inoculated into 5 mL of LB bacterial broth medium for cultivation. Following a 12 h incubation period at 37 °C with continuous agitation at 200 rpm, both bacterial species were harvested during their exponential growth phase and preserved at 4 °C for subsequent analyses.

### 2.7. Anti-Biofilm Performance Testing and Detection of Silver Ion Release

*Staphylococcus aureus* and *E. coli* were cultured in LB liquid medium on a shaker (37 °C, 120 rpm) for 24 h. The resulting bacterial solutions were then diluted 1:100, thoroughly mixed, and applied to untreated gauze and gauze modified with PDA–Ag in 24-well bacterial culture plates (1 mL per well). The plates were sealed and incubated on a shaker at 37 °C for an additional 24 h. Following incubation, the sample surfaces were washed with sterile PBS buffer to remove non-adherent bacteria, and the stained surfaces were examined microscopically. Subsequently, 500 μL of 0.1% crystal violet solution was applied to each well for 15 min, after which the samples were observed under a microscope post-treatment. The samples were extensively rinsed with sterile phosphate-buffered saline (PBS) until no visible coloration remained in the wash solution. The specimens were then air-dried on a pristine surface for 45 min. Subsequent treatment with a 30% glacial acetic acid solution for 15 min was performed to eliminate any biofilm. The optical density at 590 nm (OD590 nm) was measured using a 3 mL cuvette to quantify the biofilm content. A higher OD value indicated a greater biofilm presence, as evidenced by the removal of more biological membranes during the glacial acetic acid wash, signifying increased biofilm formation on the surface. The gauze was immersed in deionized water for 12, 24, and 36 h consecutively. Following this, a precise amount of aqueous 2-methylimidazole was added, and the advancement of the reaction was observed by measuring the optical density at 460 nm (OD 460 nm) using a 3 mL cuvette. This measurement helped quantify the silver ion concentration, with a higher OD value suggesting the formation of more complexes between the silver ion and 2-methylimidazole.

### 2.8. Antibacterial Performance Test Experiment

The gauze samples were cut into 1 cm × 1 cm sections and incubated at 37 °C for 5 h in a 100-fold diluted solution of *S. aureus* and *E. coli* (1 mL per well). Following incubation, the samples underwent three rounds of washing with a 0.9% sodium chloride (NaCl) solution. After centrifugation at 4000 revolutions per minute for 15 min, the bacteria were separated and settled. Subsequently, the resuspended bacterial solution was plated on LB agar and incubated at 37 °C for 24 h. Additionally, a 1000-fold dilution of the resuspended bacterial solution was prepared and spread on LB solid medium. The assessment of antibacterial efficacy was conducted using the following formula:

$$E=\left[\left(N_{1}-N_{2}\right)/N_{1}\right]\times 100\%$$

*E* represents the effectiveness against bacteria, *N*_1_ is the bacterial count on untreated gauze, and *N*_2_ is the bacterial count on gauze treated with PDA–Ag.

The fabric was exposed to a solution containing *S. aureus* and *E. coli* bacteria for 24 h at 37 °C. Following the removal of unattached bacteria, the gauze samples were thoroughly cleaned with a sterile PBS solution. Subsequently, the samples were immersed in a 5% glutaraldehyde solution for 12 h and stained with propidium iodide dissolved in sterile PBS solution at a concentration of 50 µg/mL for 30 min. This staining procedure was carried out in a dark environment. Post-cleaning, the samples were stored in darkness for subsequent confocal laser scanning microscopy (CLSM ) observation. Bacterial coverage on the specimens was quantified using ImageJ software (v1.8.0.345) to assess the antibacterial efficacy of the dressing.

### 2.9. Cell Compatibility Evaluation

The PDA and PDA–Ag with human cells were assessed by culturing them with cells for 8 h using a CCK-8 assay kit. Human renal epithelial cells (293T) were utilized for the toxicity evaluation of PDA and PDA–Ag on human cells. The cell culture medium containing a concentration of 2000 cells/mL was dispensed into a 96-well plate (100 µL per well) and placed in a humidified incubator for 24 h at 37 °C with a 5% CO_2_ atmosphere. Subsequently, 10 µL per well of PDA–Ag and PDA solutions at various concentrations (0, 2, 4, 8, 16, and 32 µg/mL) were added to the cell culture medium for an 8 h incubation period. Each concentration group included three replicate wells, with an additional blank control well devoid of cells. Following this, CCK-8 solution was added, and the cells were co-cultured for a specified duration before measuring the absorbance at 450 nm using an enzyme-linked immunosorbent assay (ELISA) reader. The data were processed using Nano Measure software (1.2.0.5), ImageJ software (v1.8.0.345), and Origin software (9.5.1.109).

## 3. Results and Discussion

### 3.1. PDA Surface Topography Analysis

**Figure 2:**
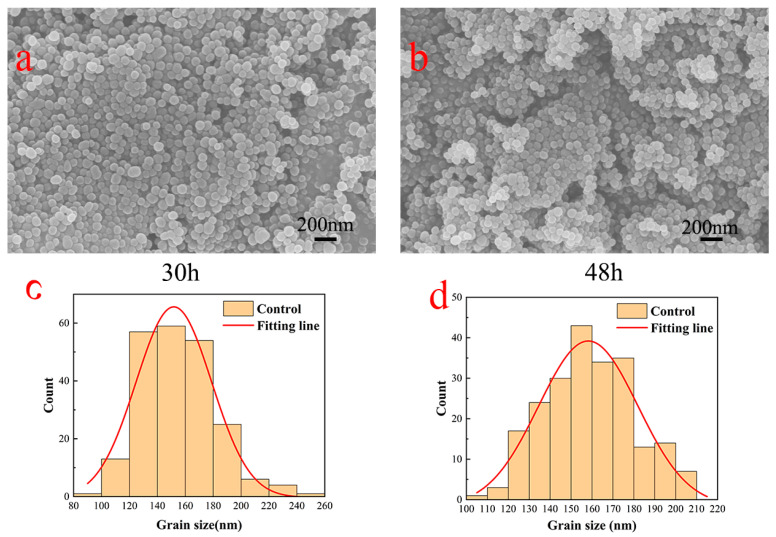
Scanning electron micrographs of particles formed after 30 and 48 h of dopamine polymerization (**a**,**b**) and particle size distributions of particles formed after 30 and 48 h of dopamine polymerization (**c**,**d**).

Dopamine underwent oxidation and self-polymerization at room temperature within an alkaline water–ethanol system to fabricate polydopamine submicron particles, as illustrated in Figure 2. SEM was employed to capture images of polydopamine submicron particles post-polymerization periods of 30 and 48 h, followed by particle size distribution analysis using Nano Measure software (1.2.0.5). It was observed that the particle size of polydopamine submicron particles was influenced by the ammonia to ethanol ratio, as reported in previous studies [18,19]. In the fabrication of dopamine submicron particles, a comprehensive investigation of multiple parameters was conducted to yield polydopamine submicron particles with enhanced uniformity and reduced size variability. Polydopamine synthesized over 30 h exhibited a predominant size distribution around 150 nm, while that synthesized over 48 h showed a slight increase in size with a main distribution peak at 160 nm. The ratio of ammonia to alcohol and the drying methodology exerted a more pronounced influence on the particle size of polydopamine compared with the reaction duration. Notably, when the ratio of ammonia to ethanol was maintained at 1:20 and freeze-drying was employed as the drying technique during polydopamine synthesis, the resulting particle size was notably smaller and exhibited greater uniformity. By considering the collective impact of various factors on polydopamine size, the optimal synthesis conditions were identified as 30 h of reaction time and 2 mL of ammonia.

### 3.2. Surface Morphology Analysis of PDA–Ag-Modified Medical Gauze

**Figure 3:**
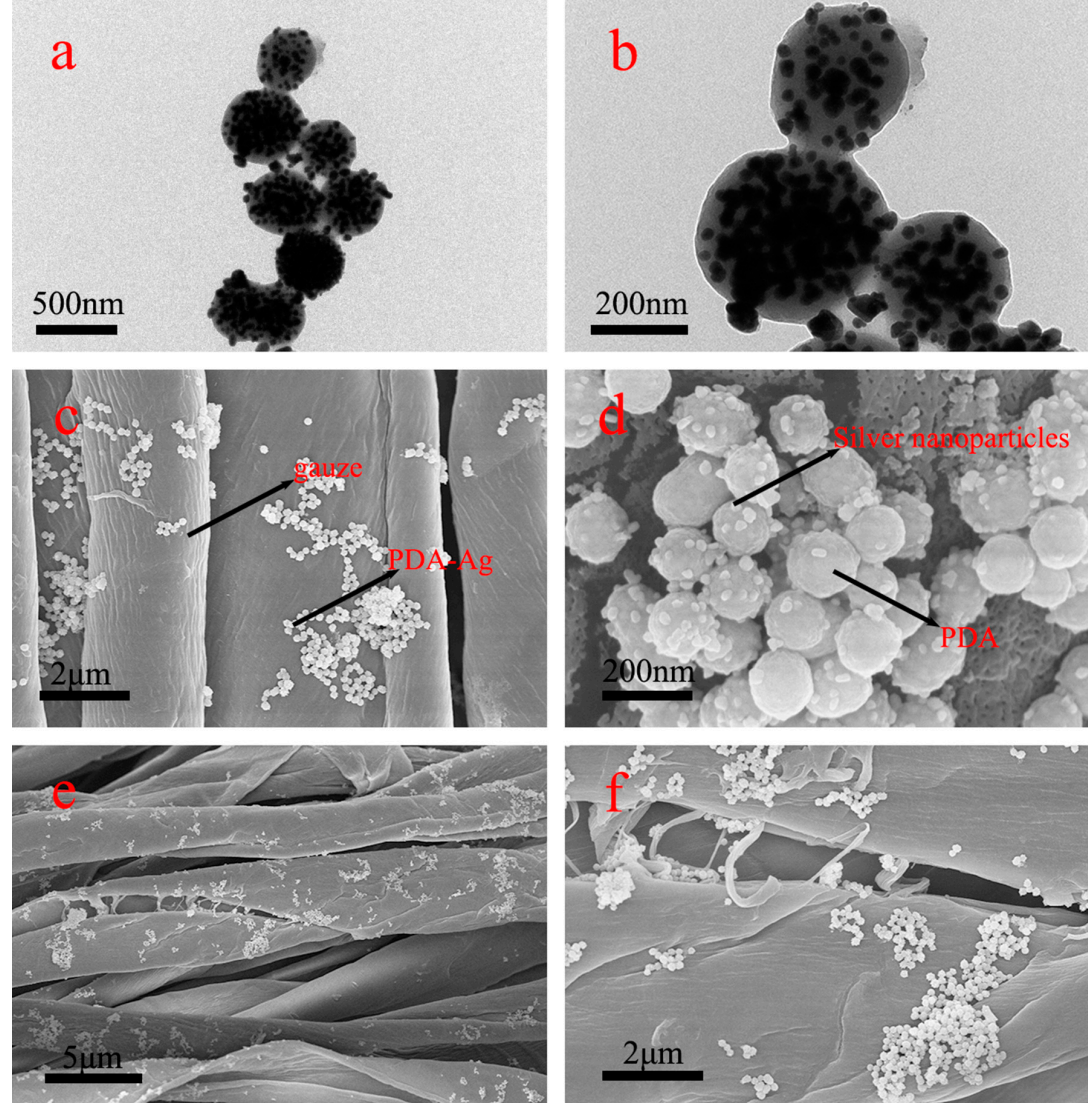
Images (**a**,**b**) were conducted using a transmission electron microscope (TEM) on PDA–Ag. The use of a scanning electron microscope (SEM) revealed the imaging of modified medical gauze. The modified gauze was observed immediately after modification (**c**,**d**) and after 48 h of modification (**e**,**f**).

**Figure 4:**
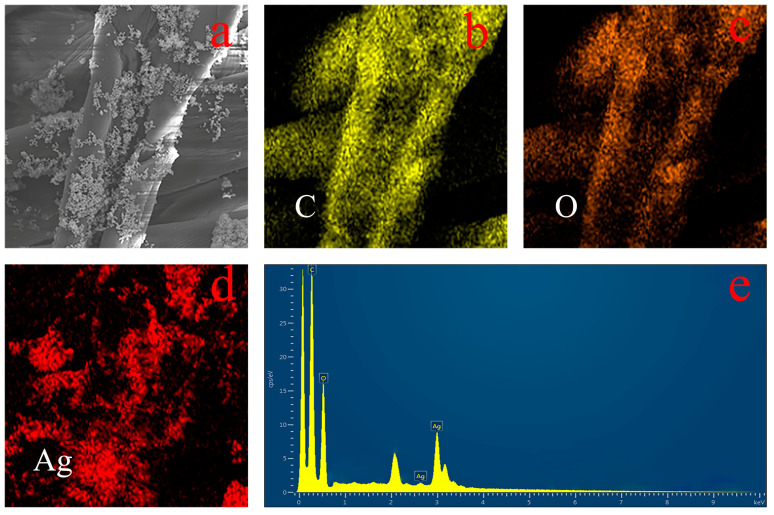
EDS of PDA–Ag on gauze surface. Image (**a**) was a scanning electron microscope image of the PDA-Ag loaded gauze, image (**b**) was the distribution of the corresponding carbon element, image (**c**) was the distribution of the oxygen element, image (**d**) was the distribution of the silver element, and image (**e**) was the distribution of the content of each element.

The PDA dispersion system was incorporated into a silver nitrate solution and subjected to ultrasonic treatment within an ice water bath. This ultrasonic ice water bath treatment effectively reduced the solubility of silver nitrate, promoting the uniform modification of silver nanoparticles within the polydopamine matrix and preventing their aggregation. Notably, during the silver ion reduction process, the polydopamine surface exhibited a pronounced affinity for silver nanoparticles. The TEM and SEM images depicted in Figure 3a–d showcase the firm attachment of silver nanoparticles to the polydopamine surface, presenting a consistently distributed spherical morphology. This configuration effectively impedes the aggregation and growth of silver nanoparticles. Furthermore, Figure 3c illustrates the successful attachment of PDA–Ag to medical gauze. Subsequently, PDA–Ag was deposited onto sterile medical gauze for 6 h, dried, and subjected to energy-dispersive X-ray spectroscopy (EDS) surface scanning. As depicted in Figure 4, the distribution of carbon, oxygen, and silver elements is discernible, with silver elements primarily localized on the gauze. Figure 4e provides insights into the elemental content on the gauze, confirming the successful introduction of silver elements. Building upon the earlier TEM and SEM analyses, it can be inferred that PDA–Ag nanoparticles are securely affixed to the gauze. Examination of the modified gauze over time using a scanning electron microscope (Figure 3e,f) revealed the persistent presence of silver nanoparticles on the PDA surface.

### 3.3. Infrared Analysis of PDA and PDA–Ag Surfaces

**Figure 5:**
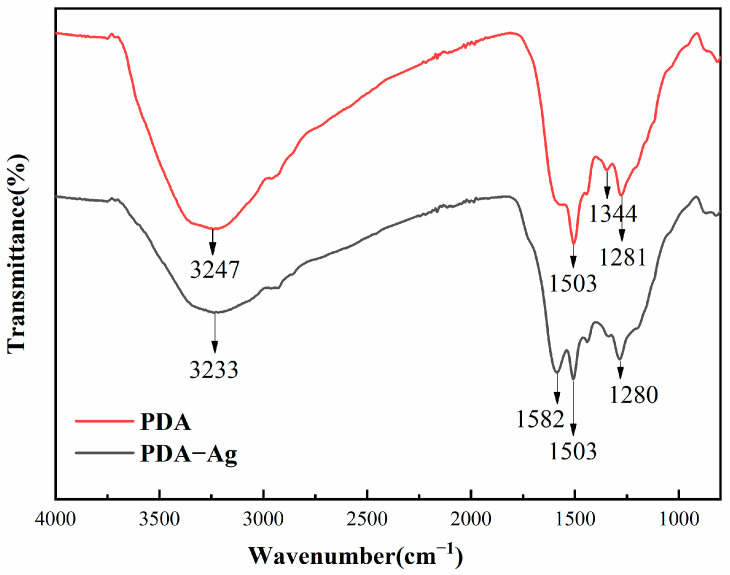
FTIR spectra of PDA and PDA–Ag surfaces.

Fourier-transform infrared (FTIR) spectra were utilized to analyze the surface chemical composition of PDA–Ag and elucidate the binding interactions between PDA and PDA–Ag, as depicted in Figure 5. Notably, the surface properties and configuration of PDA remained unchanged during the synthesis of PDA–Ag. The tensile vibration of O-H and N-H in PDA could be observed at 3247 cm^−1^. In addition, the low-frequency vibration of PDA–Ag extended to 3233 cm^−1^. The observed vibration was primarily attributed to the hydrogen bonding between the PDA and the silver nanoparticles. A broadening of the peak corresponding to C-O bonding formation was observed at 1281 cm^−1^, with a gradual shift in wavenumber to 1280 cm^−1^, suggesting alterations in the molecular environment and potential interactions within the PDA–Ag composite. In contrast to PDA, PDA–Ag exhibited an absorption band at 1582 cm^−1^, attributed to the N=O stretching vibration. Furthermore, the oxidation of catechol and hydroxyl groups led to the generation of quinone and carbonyl compounds, respectively. Concomitantly, Ag^+^ was reduced to Ag, indicating a redox reaction within the PDA–Ag composite system [20]. Simultaneously, this interaction promoted the anchoring of silver nanoparticles onto the PDA surface, thereby improving their stability within the PDA matrix. Moreover, the positively charged residual PDA may impede the aggregation of silver nanoparticles by inducing mutual electrostatic repulsion, leading to a more homogeneous dispersion. The precise mechanism underlying the adhesion of PDA–Ag composites to medical gauze remained unclear and could potentially involve interactions such as polydopamine adhesion and hydrogen bonding.

### 3.4. XPS Analysis of PDA–Ag and PDA

**Figure 6:**
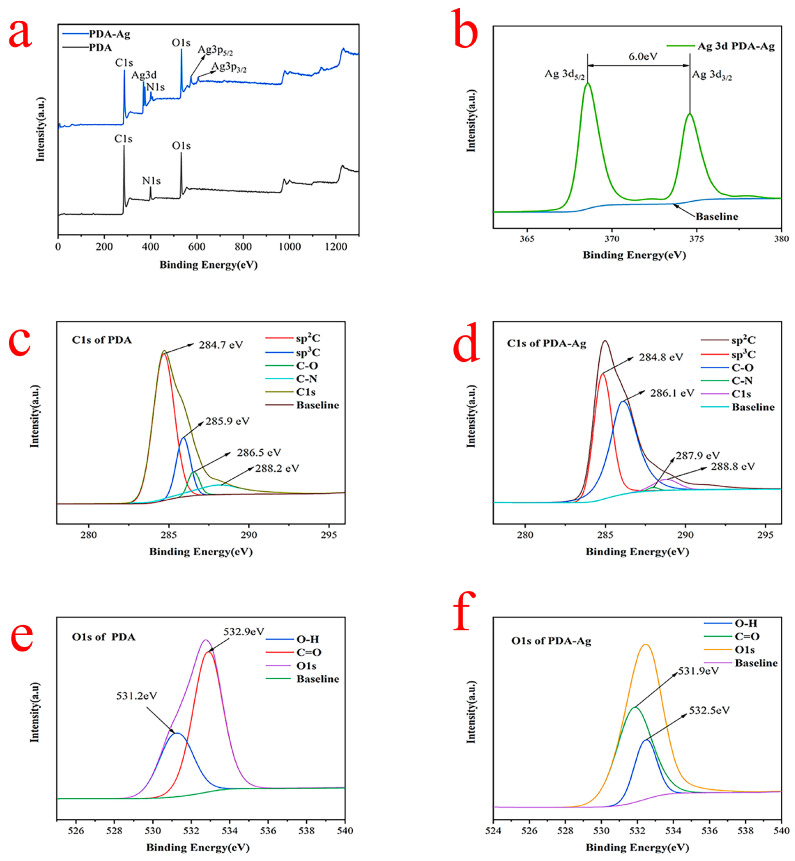
XPS of scan survey (**a**), Ag3d (**b**), C1s (**c**,**d**), and O1s (**e**,**f**) of PDA and PDA–Ag.

In Figure 6a, the XPS wide scan spectrum of PDA in the absence of silver nanoparticles revealed prominent peaks corresponding to carbon (C1s) and oxygen (O1s) signals, with relatively subdued signals observed for nitrogen (N1s). The presence of PDA nanoparticles was confirmed by the emergence of signal peaks corresponding to oxygen (O1s) and nitrogen (N1s), indicating the presence of catechol and amino groups within them. Additional signal peaks, including Ag3d and Ag3p (Ag3p5/2 and Ag3p3/2), were observed in the XPS spectrum of PDA–Ag nanocomposites, alongside the typical C1s, N1s, and O1s peaks. Notably, the Ag signal exhibited a peak at approximately 370.0 eV, confirming the presence of silver in the PDA–Ag matrix. Upon closer inspection of Figure 6b, distinct peaks for Ag3d were observed, as depicted in the figure. The two peaks were observed at 368.6 eV and 374.6 eV, with a spin-orbit separation of 6.0 eV, corresponding to the binding energies of Ag3d5/2 and Ag3d3/2, respectively. This distinct observation provided compelling evidence for the presence of elemental silver, suggesting that PDA could reduce silver ions into silver nanoparticles [21,22].

The high-resolution C1s spectrum of PDA (Figure 6c) was analyzed using a curve-fitting approach with four peak components, revealing a C1s binding energy of 284.7 eV. The binding energies for the various components were determined as follows: C 285.9 eV, C-O 286.5 eV, and C-N 288.2 eV. On the other hand, the C1s spectrum of PDA–Ag (Figure 6d) exhibited similar peak components to PDA, albeit with slightly shifted binding energies of 284.8 eV, 286.1 eV, 287.9 eV, and 288.8 eV. The notable reduction in the C-O peak intensities suggested that several oxygen-containing functional groups may have been chemically altered or removed due to interactions between the catechol groups and silver ions [23].

Figure 6e,f illustrate the high-resolution O1s spectra of PDA and PDA–Ag, respectively. Both spectra exhibited two distinct oxygen species. A peak at 531.2 eV corresponded to quinone oxygen or carbonyl (C=O) oxygen, while another peak at 532.9 eV was associated with oxygen or the hydroxyl group (OH) present on the catechol surface. A notable distinction between the O1s spectra of PDA and PDA–Ag was observed as the two peaks in the PDA spectrum shifted from 532.1 eV and 532.9 eV to 531.9 eV upon the stirring of PDA and AgNO_3_. Oxygen functional groups were prevalent in activated carbon, facilitating metal ion adsorption through ion exchange reactions. Furthermore, atomic oxygen could traverse the silver lattice with minimal energy expenditure. The robust adhesion between silver nanoparticles and the PDA surface, stemming from their interactive bond, holds significant promise and practical relevance for various applications [24].

### 3.5. Antimicrobial Properties of PDA–Ag Dressings and Detection of Silver Ion Release

The antimicrobial efficacy of PDA–Ag was investigated in this study, focusing on its antibacterial properties. The effects of PDA–Ag dressings were assessed through a comprehensive evaluation against two bacterial strains, *E. coli* and *S. aureus*.

**Figure 7:**
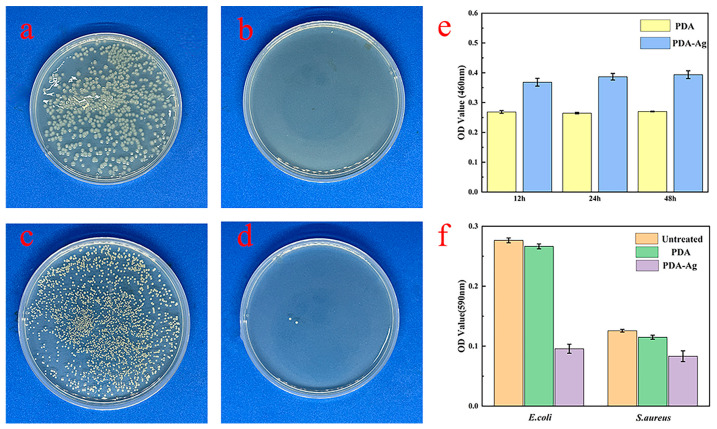
Results for *E. coli* after 5 h: (**a**) untreated sample and (**b**) PDA–Ag-loaded gauze sample. Results for *S. aureus* after 5 h: (**c**) untreated sample and (**d**) PDA–Ag-loaded gauze sample. (**e**) OD values of the complex produced with 2-methylimidazole by immersing the PDA–Ag-loaded gauze for different periods. (**f**) OD values of the acetic acid-eluting solution in the crystal violet staining assay.

After incubating dressings treated with PDA–Ag with *E. coli* and *S. aureus* for 5 h, the resulting resuspension was spread on an LB agar medium, as depicted in Figure 7. Following a 24 h incubation period, the total colony count and individual colony numbers were quantified. The antibacterial efficacies was determined to be approximately 99.99% and 99.8%, underscoring the potent antibacterial activity of the dressing against bacterial proliferation. The absorbance at 590 nm (OD value) was directly correlated with biofilm quantity, revealing a significant reduction in bacterial adsorption upon PDA–Ag treatment (Figure 7e). The antimicrobial performance of the dressing exhibited variability across different bacterial strains, exhibiting a more pronounced inhibitory effect on Gram-negative bacteria while maintaining broad-spectrum antimicrobial capabilities. This highlighted the biofilm-resistant properties of PDA–Ag-treated dressings [25,26]. The OD value of the solution increased gradually as the immersion time was prolonged, as illustrated in Figure 7e. This indicated that over time, silver ions were released to act in conjunction with the silver nanoparticles in exerting a bactericidal effect. The rate of increase in OD value slowed down, suggesting that the loading of PDA–Ag on medical gauze had been optimized to a certain extent, effectively addressing the issue of silver nanoparticle leaching.

**Figure 8:**
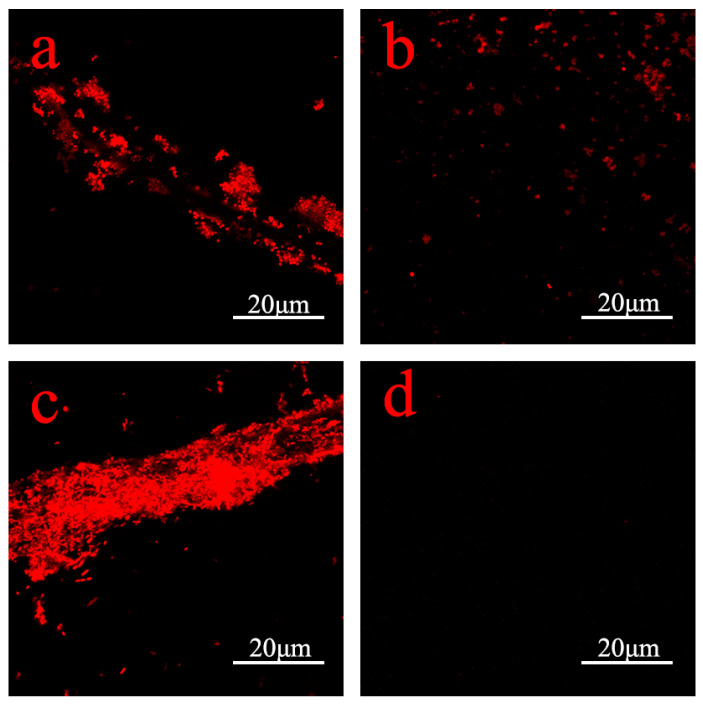
CLSM images of *S. aureus* on PDA (**a**) and PDA–Ag (**b**) modified surfaces and *E.coli* on PDA (**c**) and PDA–Ag (**d**) modified surfaces.

Bacterial attachment rates on various samples and the evaluation of their surfaces were assessed through confocal laser scanning microscopy (CLSM) images [27,28]. Analysis of the CLSM images revealed a substantial reduction in bacterial attachment on gauze modified with PDA–Ag (Figure 8b,d) compared with gauze modified with PDA (Figure 8a,c). Notably, the modified gauze exhibited superior efficacy in inhibiting Gram-negative bacteria. To quantify the antimicrobial effect of the PDA–Ag dressing, bacterial coverage on the gauze was determined using ImageJ software (v1.8.0.345). The adhesion rate of Staphylococcus aureus decreased from 6.284% to 2.263%, resulting in an antibacterial rate of 63%. Similarly, the adhesion rate of Escherichia coli decreased from 20.557% to 0.017%, achieving an impressive antibacterial rate of 99.91%. The bacteriostatic impact of silver nanoparticles primarily stems from their interactions with bacterial enzymes, proteins, and/or the disruption of bacterial membranes [29]. Statistical analysis of the adhesion rates of both bacterial strains demonstrated that PDA–Ag significantly reduced bacterial adhesion, affirming the robust antimicrobial performance of the PDA–Ag-modified surface.

### 3.6. Cell Compatibility Evaluation

**Figure 9:**
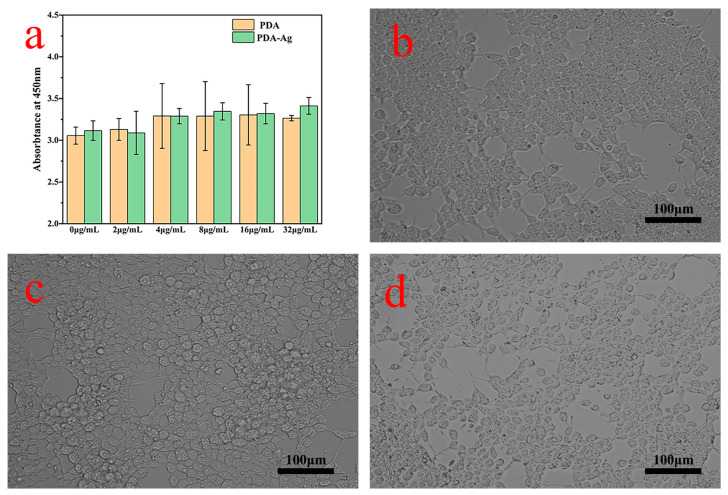
Cellular toxicity tests (**a**) and cell morphology of untreated (**b**), PDA-added (**c**), and PDA–Ag-added cells (**d**).

The assessment of cell adhesion and proliferation characteristics in solution serves as a valuable method for evaluating sample toxicity to cells [30,31]. The measurement of absorbance at 450 nm offers insights into cell concentration, facilitating the comparison of relative cell viability based on absorbance readings. The CCK-8 assay results revealed a slight increase in cell absorbance (OD) values with escalating concentrations of PDA and PDA–Ag (Figure 9a), suggesting that both PDA and PDA–Ag did not impede cell growth and facilitated cell proliferation to some extent. Notably, Figure 9b–d illustrated that human renal epithelial cells exhibited strong adhesion and maintained normal morphology following an 8 h co-cultivation with PDA and PDA–Ag. Furthermore, their survival rates exceeded 90%, underscoring the excellent cell compatibility of the PDA and PDA–Ag composite material [32].

## 4. Conclusions

The study meticulously immobilized silver nanoparticles onto polydopamine surfaces and fine-tuned the particle size of polydopamine nanoparticles. The synthesis of PDA–Ag was achieved in a single step without the generation of additional byproducts, thereby exemplifying an environmentally friendly approach. The formation process of PDA–Ag composite materials was comprehensively investigated using a combination of SEM-EDS, TEM, and FTIR analyses. The synthesized PDA–Ag composite material was effectively immobilized on the surface of medical gauze, demonstrating strong adhesion and uniform distribution. Through in vitro antibacterial and cytotoxicity experiments, the adhesion rate decreased by 63% and 99.91%, respectively. The antibacterial efficacy of the dressing was evaluated by diluting the co-cultured bacterial solution and applying the dressing. After a 24 h incubation period, the number of colonies was quantified. The findings revealed remarkable antimicrobial efficacies of 99.99% and 99.8% for the dressing. Additionally, the dressing demonstrated the ability to inhibit biofilm formation. Furthermore, cell co-culture experiments demonstrated the excellent biocompatibility of PDA–Ag with human cells. The release of silver nanoparticles from medical gauze was enhanced by leveraging the distinctive structure and characteristics of polydopamine-loaded nanosilver to enhance the antimicrobial efficacy of silver nanoparticles. The findings suggest that PDA–Ag-modified gauze holds significant promise for clinical anti-infective wound treatment. Subsequent investigations will concentrate on leveraging this formulation to enhance wound healing in vivo, optimizing the uniform dispersion of PDA–Ag on gauze, and evaluating the antimicrobial activity of polydopamine nanosilver at varying concentrations, crucial for clinical translation.

## Data Availability

The raw data supporting the conclusions of this article will be made available by the authors on request.
